# Wrist Stabilising Exercise Versus Hand Orthotic Intervention for Persons with Hypermobility – A Randomised Clinical Trial

**DOI:** 10.1177/02692155241293265

**Published:** 2024-10-29

**Authors:** Lindholm Susanne, Claesson Lisbeth

**Affiliations:** 1 Registrated Occupational Therapist, Master of Science (MSc), 195564 Institute of Neuroscience and Physiology, University of Gothenburg, Gothenburg, Sweden; 2 Associate Professor, Registrated Occupational Therapist, Department of Health and Rehabilitation, 195564 Institute of Neuroscience and Physiology, University of Gothenburg, Gothenburg, Sweden

**Keywords:** Hypermobility Spectrum Disorder, hypermobility Ehlers Danlos Syndrome, activity ability, pain, paraesthesia

## Abstract

**Objective:**

To investigate the effectiveness of wrist stabilisation exercises compared to conventional intervention, whether it reduces pain and/or paraesthesia in the hand, as well as how the interventions affected activity ability, health-related quality of life and effects on hand function and grip strength in people with Hypermobility Diagnosis.

**Design:**

A randomised controlled trial.

**Setting**: Units of Occupational therapy in Primary Care, Kalmar County Council, Sweden

Participants: The study included 169 participants’ data randomised to the Exercise group (n = 83) or the Control group (n = 86). The samples consisted of adults in diagnosed Hypermobility Spectrum Disorders or hypermobility Ehlers Danlos Syndrome with symptoms of pain and/or paraesthesia in the hands in the last three years.

**Interventions**: The Exercise group trained according to structured progressive exercises and weights programme. The Control group used the hand orthosis during selected activities. Both groups performed randomised intervention for 12 weeks.

**Main measures**: The primary outcome was the Disabilities of Arm, Shoulder, and Hand questionnaire. Secondary outcomes were the Grip Ability Test, the Jamar dynamometer and the EuroQol EQ-5D.

**Results:**

There were 116 subjects who completed the intervention. There were no statistically significant difference between the wrist stabilisation exercise and the conventional intervention in terms of activity ability, health-related quality of life, hand function, grip strength, pain or paraesthesia in people with Hypermobility Spectrum Disorders or hypermobility Ehlers Danlos Syndrome.

**Conclusion:**

There is no statistically significant difference between the Exercise group and the Control group regarding activity ability after 12 weeks intervention period.

## Introduction

Everyday life is affected by the function of the hand. Hand function includes the ability and skill to perform a single task, but also based on coordination and grip.^1^ Hypermobility Spectra Disorders is a condition that can cause the joint to move outside the normal range expected for a specific joint, but also includes skin fragility and elasticity, joint instability and/or weakness in muscles, ligaments, and connective tissue.^2^ Ehlers-Danlos syndrome is a heterogeneous group of connective tissue disorders characterised by musculoskeletal, skin, and vascular manifestations and consists of 13 different variants, with hypermobility Ehlers-Danlos syndrome clearly dominating (80–90%).^3^ Symptoms often start at a young age. The prevalence varies and is found in approximately 10–20% of the world’ population. From a gender perspective, hypermobility is more common in women (70–80%) than in men and decreases with age.^3^ In the present study, Hypermobility Spectrum Disorders and hypermobility Ehlers-Danlos syndrome are considered as synonymous and are hereafter referred as Hypermobility Spectrum Disorders/hypermobility Ehlers Danlos Syndrome. A laxity of the ligaments, for example in the wrists and fingers, can contribute to weakness and joint instability, which increases the risk of injuries in connection with occupation.^4^ Sustained loading in end position also increases the risk of injury and can lead to pain, paraesthesia or sub-/dislocation in the hand.^1,5^ Proprioception has the task of sensing the position of the joint, with what speed and precision the hand needs to move and with what force is required to perform an activity or movement.^6^ Activities performed in the same (static) position can contribute to a feeling of stiffness, even if the joint itself is hypermobile. It has also been noted that manual activities that require repetition or sustained positions are difficult, as are those that require grip strength.^4^ Instability and pain in the wrist affect activity ability, which can also affect health-related quality of life. There are some research showing that hypermobility causes difficulties in performing everyday tasks due to symptoms from the wrists and fingers, as well as pain or paraesthesia in the hands^.1,7^ A study investigated an occupational therapy intervention to determine whether hand orthosis affects handwriting endurance, grip strength, or self-perception of writing,^8^ but we found no scientific studies that have examined the outcome of stabilising exercise for wrists in people with hypermobility diagnoses. There is a lack of robust research on improved hand function and grip strength, and how activity ability and health-related quality of life are affected by hand rehabilitation for people with Hypermobility Spectrum Disorders/hypermobility Ehlers Danlos Syndrome.

The aim of this trial is to investigate the effectiveness of wrist stabilisation exercises compared to conventional intervention including a hand orthoses, in people with Hypermobility Spectrum Disorders or hypermobility Ehlers Danlos Syndrome with the intention of reducing pain and/or paraesthesia in the hand. In addition, the study examines how the intervention affects the activity ability, hand function, grip strength and health-related quality of life.

## Methods

This study was a prospective randomised controlled trial, following the CONSORT guidelines.^9,10^ The intervention, Exercise group, was compared with the Control group, a conventional intervention using a hand orthosis. The study was registered on ClinicalTrials.gov with the identifier: NCT05696041, where a trial protocol is available. The study is reported according to the Consolidated Standards of Reporting Trials (CONSORT) for randomised trials.^11^ Participants were recruited from the journal system Cambio Cosmic in Kalmar County Council, Sweden. The study has followed ethical guidelines and regulations according to the Declaration of Helsinki.^12^ The Ethical Review Board in Linkoping, Sweden approved the study (Dr#2014/133–31). The inclusion criteria consisted of Swedish speaking adults (> 18 year), who have/have had intermittent or persistent pain and/or paresthesia in one or both hands during the last three years. They needs to be diagnosed with Hypermobility Spectrum Disorders M 35.7 or Ehlers Danlos Syndrome Q79.6, according to ICD-10-SE, during the period 1 January 2008 to 31st December 2013.^13^ Participants were excluded if they suffered another illness or injury, such as stroke, fracture or injury in the arm or hand, during the past 6 months, that could have affected the study.

Participants were informed about the study in writing by a letter, and they returned a signed consent form. A code list was created to randomly assign participants to eighter the Exercise group or the Control group by a statistician. The authors had no physical contact with the participants but prepared all materials for the intervention, except for the orthoses, which were prescribed by Occupational therapists. Approximately 20–25 Occupational therapists with experience of hand therapy at the Primary Care units (12 in number) attended a half-day training session by the first author to ensure that they carried out interventions and documentation as equally as possible. They received a manual and information about the forms, measuring instruments and how to complete the study protocol. The Occupational therapists contacted the participants who visited the Primary Care units, for measurements and interventions. Neither Occupational therapists nor participants knew which intervention the participants were randomised to until the participants first visit to the Primary Care unit, which stated the baseline. The randomised intervention began and 3–4 follow-up sessions of the exercise programme were planned. Participants were given an exercise diary during their first visit. Furthermore, the Occupational therapists collected basic demographic data, such as age, work/employment, education level and gender, which were documented in the study protocol. The Occupational therapists wrote medical journal entries according to standard procedures. No compensation was provided to the participants. Data collection took place from 1 August 2014 to 31 December 2014.

The primary outcome was the Disabilities of Arm, Shoulder, and Hand questionnaire,^14,15^ a self-report instrument measuring the ability to perform daily activities involving the shoulder, arm and hand. This questionnaire has three parts, and part 1, was used in this study. Participants rated their ability to perform activities on an ordinal scale from 1 (no difficulty) to 5 (impossible to do). A maximum of three missing answers were allowed in the questionnaire. A total score was ranging from 0 (no disability) to 100 (severest disability). The questionnaire has been translated into Swedish and tested for reliability and validity.^14,15^ In this study, it was used as a measure of activity ability, and also pain in general and pain during activity (items 24, 25) and finally paraesthesia (item 26). It has been suggested that a ​​change of more than 10 points on the Disabilities of Arm, Shoulder, and Hand score, for illnesses/injuries of the upper limb, would be regarded as clinically significant.^13^

Additional outcomes included the Grip Ability Test,^16^ which measures hand function through three sub-tasks: putting on a flexi grip sock over the non-dominant hand, picking up a paper clip and attach it to an envelope, and finally filling a glass of water from a jug (1 litre of water). The test is scored based on a formula, with a normal range between 11–20 (mean 16.5). Scores above 20 indicate impaired hand function while scores below 11 indicates better hand function than normal.^16^ The Grip Ability Test is valid, sensitive to change, and with intraobserver (r = 0.99) and interobserver (r = 0.95).^17^ Hand strength (grip) was measured using a Jamar dynamometer, with tree measurements taken for each hand. The average of these measurements was recorded in kilograms (with 1 decimal).^18^ The measurement has been translated into Swedish and tested for reliability and validity.^19^ The final outcome measured was the EuroQol EQ-5D, a self-report measurement for health-related quality of life. This study used perceived health status on a visual analogue scale from 0 (worst possible health status) to 100 (best possible health status).^20^

The Exercise group received exercise programmes and written information, designed by the Hand and Plastic Surgery Rehabilitation Unit at Linköping University Hospital, Sweden. The programme was a structured method with progressive exercises and weights. They started the exercises with 3 × 10 repetitions which could be increased up to 8 × 10 repetitions. Starting weight from 0.5 which could be increased to up to 1.0 kg. The intensity was tailored and graded according to the person's needs. Participants were instructed to perform the exercises daily for 12 weeks (Supplementary material 1 and 2). The occupational therapists documented changes and deviations in the study protocol. The participants also registered their progress in a training schedule.

The Control group (conventional intervention) received hand orthosis aimed at relieving pain, or stabilisation of the wrist. Each participants in this group was prescribed an orthosis with a volar plastic or metal splint for one or both wrists depending on their needs to prevent loading in end position, flexion, or extension of the wrist. The orthosis was to be used during such as, sleeping, vacuuming, carrying, washing, cycling, or driving.^8,21^ Participants were instructed to keep a daily of log how and when they used (or did not use), the orthoses during the 12-week intervention.

Both, the Exercise group and the Control group received general information about hand anatomy, physiology, and hand function in daily activities. They were also given advice on how they can perform daily tasks more effectively based on ergonomic principles.^1,7,22–24^

The statistical analyses were conducted in the software Statistical Package for the Social Sciences (SPSS), version 22 IBM Corp. Released 2013. (IBM SPSS Statistics for Windows, Version 22.0. Armonk, NY: IBM Corp)^25^ and in the R statistical environment.^26^ A Power analysis (ANOVA analogous Student's t test) was used to calculate the size of the sample. We assumed that if a change of 20%, i.e., at least 10-point difference in the Disabilities of Arm, Shoulder, and Hand score,^14^ as clinically significant and that a power of 80% is required, then the study will need 60 persons per group when the cohort is normally distributed. For all tests, we used a significance level of 0.05.^9,26,27^ This study used means (*M*), median (*Md*), interquartile range (*IQR*) and standard deviation (*SD*) for descriptive statistics as age, or when more than one answer option was available for the same question, for example the Disabilities of Arm, Shoulder and Hand. EuroQol EQ-5D health-related quality of life (EuroQol EQ-5D the self-assessment of perceived health status) used percentage (%).

To test whether the observed differences in the change of activity ability of the Disabilities of Arm, Shoulder, and Hand score, hand function the Grip Ability Test and grip strength, and finally the EuroQol EQ-5D health-related quality of life scores between the participants who were allocated to wrist stabilising exercises and those who received the conventional intervention were statistically significant, we conducted a series of independent samples *t*-tests. The analysis followed the principles for intention-to-treat.^9,27^ The participants began the allotted intervention, and no change of intervention group took place. Similarly, to test whether the observed differences in the change of item 24, 25 and 26 in Disabilities of Arm, Shoulder, and Hand scores between the participants who were prescribed wrist stabilising exercises and those who received only the hand orthotic intervention were statistically significant, we conducted a series of Wilcoxon rank-sum tests.^26^ The training diaries were categorised based on the number of training sessions per day and week, performed by the participants in both the Exercise group and the Control group. The data are presented descriptively with the number of training sessions and percentage (%).

Analysis of missing data was done by examining whether there were differences in age, gender, employment and level of education in comparison with the study groups.^9,27^ The analyses consisted of Exercise group n = 76, and Control group n = 77.

## Results

**Figure 1.**
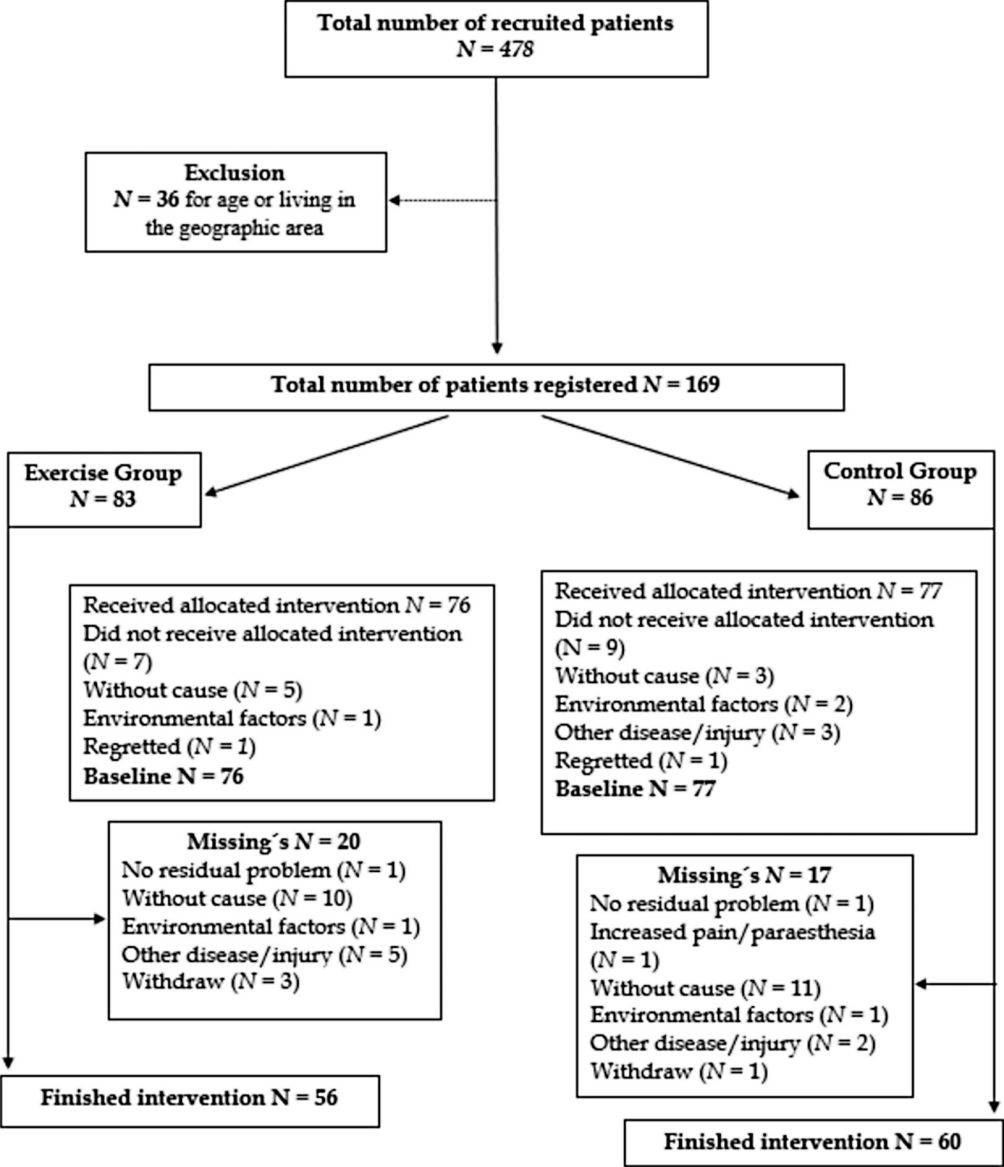
Flow chart of the participant´s progress through the study.

The study results are presented from baseline to finished intervention at 12 weeks based on actual values from the data. Flow chart (Figure 1) showed that n = 478 individuals were assessed as eligibility, and n = 169 were enrolled and allocated to Exercise group (n = 86 and) or Control group (n = 83). Demographic data, and subject characteristics explains in Table 1. Gender distribution at baseline was 74 women in the Exercise group, and 73 women in the Control group. At the end of the intervention, 116 participants remained. At the 12-week follow-up, there were missing's in the Exercise group (n = 20) and in the Control group (n = 17). Comparison of characteristics at baseline between the missing's and the study groups shows that there was no difference regarding age, gender, employment, and level of education between the groups.

**Table 1.** Baseline characteristics for the total sample grouped into exercise group and control group.

	Exercise group	Control group
Characterises	Baseline	Baseline
	*N *= 76	*N *= 77
Age ***M*** (*SD*) Age *Md* [IQR] 18–34 year (*%*) 35–44 year (*%*) 45–54 year (*%*) 55–64 year (*%*) 65–80 year (*%*)	48.5 (11.6) 49.0 [42, 57] 8 (10*%*) 21 (28*%*) 19 (25*%*) 25 (33*%*) 3 (4*%*)	48.6 (12.6) 46.0 [42, 57] 9 (12*%*) 18 (23*%*) 27 (35*%*) 14 (18*%*) 9 (12*%*)
Sex Women Men	74 (97*%*) 2 (3*%*)	73 (95*%*) 4 (5*%*)
Employment Work/study (100*%*) Sick leave (100*%*) Partly work/study/sick leave (*%*) Partly sick leave/retired (*%*) Retired (*%*) Unemployed/off duty (*%*) Missing (*%*)	12 (16*%*) 25 (33*%*) 21 (28*%*) 6 (8*%*) 11 (14*%*) 1 (1*%*)	15 (20*%*) 13 (17*%*) 25 (32*%*) 8 (10%) 10 (13*%*) 6 (8*%*)
Education Lower secondary school (*%*) Higher secondary school (*%*) University education (*%*) Missing (*%*)	11 (14*%*) 43 (57*%*) 21 (28*%*) 1 (1*%*)	9 (12*%*) 39 (50*%*) 26 (34*%*) 3 (4*%*)

Note: Interquartile range [*IQR*], Mean (*M*), Median (*Md*), Numbers (*N*), Percent (*%*), Standard Deviation (*SD*).

**Figure 2.**
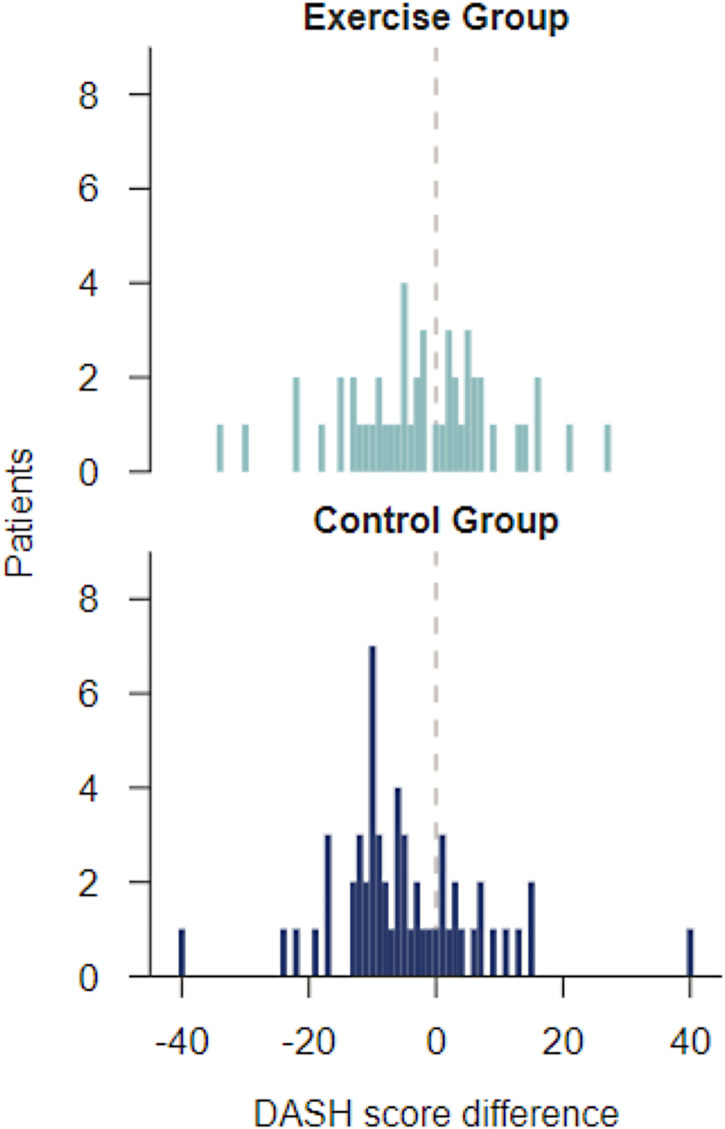
Bar chart DASH score difference from baseline and after 12 weeks (12 weeks of training).

There was no statistically significant difference between the Exercise group and the Control group at baseline or after finished intervention at 12 weeks regarding activity ability (Disabilities of Arm, Shoulder, and Hand score), hand function (Grip Ability Test), grip strength (Jamar), pain (Disabilities of Arm, Shoulder, and Hand item 24 and 25) and paraesthesia (Disabilities of Arm, Shoulder, and Hand item 26) or health-related quality of life (EuroQol EQ-5D) (Table 2). Table 3 shows effect size in activity ability, hand function and grip strength tests and EuroQol EQ-5D Health State by the Exercise group and the Control group. The results show that there are differences in the Exercise group, but the variation is large in the sample and therefore the effect size becomes uncertain (Table 3). Figure 2 shows the disabilities of arm, shoulder, and hand score difference, where the exercise group after completion of the intervention indicates a difference, but the heterogeneity in both groups demonstrates great variation.

**Table 2.** Activity ability, hand function tests and EuroQol −5D Health State by the Exercise group and the Control group at baseline and after 12 weeks.

	Exercise group	Control group	Baseline		Exercise group	Control group	12 weeks	
	Baseline *N *= 76	Baseline *N *= 77	*p*- value	CI (95*%*)	12 weeks *N *= 56	12 weeks *N *= 60	*p*- value	CI (95*%*)
DASH score								
Mean (*SD*)	46.4 (16.1)	45.8 (16.8)	0.83	−4.7, 5.8	43.0 (20.1)	40.7 (17.5)	0.51	−4.6, 9.3
Median [*IQR*]	47.5 [33.4, 60.0]	45.8 [33.0, 58.6]			44.2 [25.0, 57.7]	41.7 [27.7, 53.3]		
								
Grip Ability Test								
Mean (*SD*)	25.7 (10.1)	26.6 (9.3)	0.54	−4.1, 2.1	22.8 (10.6)	23.1 (7.3)	0.84	−3.6, 3.0
Median [*IQR*]	24.4 [19.3, 30.0]	25.2 [21.0, 30.5]			20.4 [16.5, 25.6]	22.0 [17.5, 25.9]		
								
Jamar dynamometer left hand								
Mean (*SD*)	22.7 (9.8)	23.0 (9.5)	0.86	−3.4, 2.8	24.3 (8.8)	23.5 (9.6)	0.64	−2.6, 4.2
Median [*IQR*]	22.4 [15.5, 29.9]	23.0 [18.0, 28.7]			24.0 [18.3, 29.8]	24.3 [16.5, 29.2]		
								
Jamar dynamometer right hand								
Mean (*SD*)	22.6 (9.9)	24.1 (10.1)	0.34	−4.7, 1.7	25.6 (9.6)	24.4 (10.9)	0.54	−2.6, 5.0
Median [*IQR*]	23.0 [14.6, 30.6]	24.7 [17.3, 29.8]			25.2 [19.3, 33.2]	25.7 [15.3, 30.5]		
								
EQ-5D Health State								
Mean (*SD*)	49.6 (19.7)	53.2 (22.0)	0.29	-10.3, 3.1	55.3 (20.6)	57.8 (19.7)	0.51	-9.9, 5.0
Median [*IQR*]	50.0 [30.0, 65.0]	50.0 [40.0, 70.0]			60.0 [35.0, 75.0]	57.5 [40.0, 70.0]		
								
DASH 24 Pain in general								
Mean (*SD*)	3.3 (0.9)	3.4 (0.9)	0.86		3.0 (1.0)	3.1 (0.9)	0.74	
Median [*IQR*]	3.0 [3.0, 4.0]	3.0 [3.0, 4.0]			3.0 [2.0, 4.0]	3.0 [3.0, 4.0]		
								
DASH 25 Pain in activity								
Mean (*SD*)	3.6 (0.9)	3.6 (0.9)	0.89		3.4 (1.0)	3.3 (0.9)	0.65	
Median [*IQR*]	4.0 [3.0, 4.0]	4.0 [3.0, 4.0]			3.0 [3.0, 4.0]	3.0 [3.0, 4.0]		
								
DASH 26 Paraesthesia								
Mean (SD)	3.0 (1.1)	2.9 (1.0)	0.40		2.6 (1.1)	2.4 (1.2)	0.56	
Median [*IQR*]	3.0 [2.0, 4.0]	3.0 [2.0, 4.0]			3.0 [2.0, 4.0]	2.0 [1.0, 3.0]		

Note: Confidence interval (*CI*), Standard deviation (*SD*), Numbers (*N*), Percent (*%*), Mean (*M*), Median (*M*), Interquartile Range (*IQR*), EuroQol (EQ-5D), Disabilities of the Arm, Shoulder and Hand (DASH) score, Item 24 Pain in general, Item 25 Pain in activity and Item 6 Paraesthesia.

**Table 3.** Effect size in activity ability, hand function tests and EuroQol −5D Health State by the Exercise group and the Control group from baseline and after 12 weeks.

	*Independent T- test*		
	*p*	*Estimate*	*CI 95%*
GAT score	0.63	0.64	−1.98, 3.26
Jamar left hand score	0.78	0.30	−1.82, 2.42
Jamar right hand score	0.21	1.35	−0.76, 3.46
DASH score	0.28	2.55	−2.09, 7.19
EQ-5D	0.56	-2.59	-11.42, 6.24

Notes: Grip Ability Test (GAT), Jamar (Jamar dynamometer), the Disabilities of Arm, Shoulder and Hand questionnaire (DASH), EuroQol EQ-5D health-related quality of life, the self-assessment of perceived health status (EQ-5D), Confidence Interval (CI 95%).

The training diaries (Table 4) showed that compliance in performing the exercises according to the programme was slightly higher in the Exercise group. Orthoses were used mostly at night, followed by driving a car. There were slightly fewer subjects from the Exercise group who submitted the exercise diary 26.6% compared to the Control group 33.1%. But 40% of the diaries were not submitted.

**Table 4.** Reported number of days/week in the training diary by the Exercise group and the Control group.

	Exercise group		Control group					
Days/week	Exercise (*%*)	Exercise ball (*%*)	Night (*%*)	Vacuum (*%*)	Carrying (*%*)	Laundry (*%)*	Cycling (*%*)	Driving (*%*)
6–7/7	65.6	63.7	45.8		3.4		0.8	14.6
4–5/7	15.6	15.7	18.9	2.1	8.7	1.3	4.4	20.0
2–3/7	5.7	5.9	9.7	18.9	32.6	25.8	5.3	20
0–1/7	13.1	14.6	25.7	79	55.2	73	89.5	45

Note: Percent (*%*). The exercises group kept a diary of their training sessions. In the control group the subjects were supposed to use orthoses in following activities; at night when they sleep, when they vacuum, carrying and handled the laundry, when they cycle and drive a car.

## Discussion

This randomised controlled trial examined the effectiveness of wrist stabilising exercises compared with the conventional intervention, which included a hand orthosis for people with Hypermobility Spectrum Disorders/hypermobility Ehlers Danlos Syndrome. The results showed no statistically significant improvement between the Exercise group and the Control group for activity ability, hand function, grip strength, and self-reported perceived health-related quality of life, from baseline to 12 weeks of intervention. Nor paraesthesia and/or pain showed any statistically significant differences between the Exercise group and the Control group.

There are certainly several explanations for the fact that both interventions had an equivalent effect. Pain was reported from both groups and more pain are associated with activity. This may indicate that pain is often related to loading and/or hyperextension of the wrists.^21,28^ This study also showed that paraesthesia in the hand is common in people with Hypermobility Spectrum Disorders/hypermobility Ehlers Danlos Syndrome. Paraesthesia in the present study was reduced, both the Exercise group and the Control group seem to have some effect on paraesthesia regardless of intervention. Proprioception in hypermobility often seems to be impaired, which of course affects the quality of activity ability. Persons with hypermobility describe that they fumble, that they drop things from their hands, that they often use too much force when holding things, and that they lose strength and grip during activities over time.^22^ Both the Exercise group and the Control group improved hand function and grip strength after intervention, which may indicate that targeted interventions for the hand make differences. Orthoses for pain in the wrist are a common intervention, and it is possible that the orthosis contribute to pain relief, support, stability or reduced risk of the joint ending up in end position.^29^ A study indicates that when the wrist is supported, pain in the wrist and/or fingers is reduced, which also can contribute to improved hand function, and increase the activity ability.^1^ Grip strength results showed improved strength´s at the end of the intervention for both hands for the Exercise group and the Control group. From a clinical perspective, it seems that the Grip Ability Test contribute to information and indicate how the motor skills of the hand work, and with what quality the person perform tasks with the hands. Grip strength tests such as Jamar dynamometer provide information on the strength and to some extent also the endurance to perform activities with the hands. This is important information for the occupational therapist to design tailored intervention programmes to the person's needs. In Hypermobility Spectrum Disorders /hypermobility Ehlers Danlos Syndrome, pain is common (>90%), which can be regional or general.^30^ If the person has pain in the hand at rest and/or during activity, or if there is paraesthesia in the hand intermittent or persistent, we can clinically assume that hand function and grip strength are probably affected. In other words, there may be underlying causes like pain or paraesthesia in the hand that reduced activity ability, and contribute to a poorer quality of life, an impaired autonomy and suffering for the person.

We are aware that there is a significant amount of missing data, which is a weakness that may affect the results. We sent out a consent letter, where the person himself considered whether the inclusion criteria matched their symptoms (later verified by the Occupational therapist). It is known, but not to what extent, that pain and/or paresthesia in the hand occurs in people with hypermobility spectrum disorders/hypermobility Ehlers-Danlos syndrome. We chose to include paresthesia as an inclusion criterion because it can affect the ability to perform activities. It is possible that if we had chosen a consecutive sample instead, we would have had a higher participation. It is possible that the participants felt that the interventions required more resources and time than expected. It is also possible that the duration of the intervention of 12 weeks may have some impact on the number of missing. Furthermore, if participants did not experience any improvement and therefore it was not worthwhile to continue.

There are 13 variants of Ehlers Danlos syndrome carry the same ICD-10 diagnosis code and cannot be distinguished from each other, but from a clinical perspective we lean towards the fact that 80–90% of those diagnosed with Ehlers Danlos syndrome have the hypermobile variant.^3^ From a rehabilitation perspective, the intervention is the same for both Hypermobility Spectrum Disorders and hypermobility Ehlers Danlos Syndrome. The gender distribution had a significant preponderance of women in both the training group and the control group (95–97%). It is known from previous studies that it is more common in women in adulthood.^3,31^

We cannot rule out that some participants previously received a hand orthosis and may also have used it during the intervention in the Exercise group, which may have affected the results. The assessment instruments used in this study have been tested for validity and reliability but are not specifically tested for individuals with hypermobility spectrum disorders/hypermobility Ehlers-Danlos syndrome. The instruments measure different aspects (function and activity) and are varyingly sensitive to changes. It can also be difficult to evaluate an exercise regimen when it is self-reported.^9^ We performed a variety of statistical analyses to detect, if possible, even minor differences between the training group and the Control group, but we found no differences. This may indicate significant heterogeneity in people with hypermobility diagnoses, or that the assessment instruments we used were too blunt.

The conclusion is that there is no statistically significant difference between the Exercise group with wrist stabilization training and the Control group with hand orthosis in terms of activity ability, health-related quality of life, hand function, grip strength, pain or paresthesia in people with Hypermobility Spectrum Disorders/hypermobility Ehlers Danlos syndrome. Further research is needed to investigate effective and sustainable interventions for wrists.

**Clinical message**

- There is no statistically significant difference between the Exercise group and the Control group regarding activity ability after 12 weeks intervention.
- Using wrist stabilisation exercise has the same equivalent result as orthotic treatment after a 12-weeks intervention.
- Stabilisation exercise and orthosis are relevant and useful for people with Hypermobility diagnosis.

## Supplemental Material

sj-docx-1-cre-10.1177_02692155241293265 - Supplemental material for Wrist Stabilising Exercise Versus Hand Orthotic Intervention for Persons with Hypermobility – A Randomised Clinical Trial Supplemental material, sj-docx-1-cre-10.1177_02692155241293265 for Wrist Stabilising Exercise Versus Hand Orthotic Intervention for Persons with Hypermobility – A Randomised Clinical Trial by Lindholm Susanne and Claesson Lisbeth in Clinical Rehabilitation

sj-docx-2-cre-10.1177_02692155241293265 - Supplemental material for Wrist Stabilising Exercise Versus Hand Orthotic Intervention for Persons with Hypermobility – A Randomised Clinical Trial Supplemental material, sj-docx-2-cre-10.1177_02692155241293265 for Wrist Stabilising Exercise Versus Hand Orthotic Intervention for Persons with Hypermobility – A Randomised Clinical Trial by Lindholm Susanne and Claesson Lisbeth in Clinical Rehabilitation

sj-docx-3-cre-10.1177_02692155241293265 - Supplemental material for Wrist Stabilising Exercise Versus Hand Orthotic Intervention for Persons with Hypermobility – A Randomised Clinical Trial Supplemental material, sj-docx-3-cre-10.1177_02692155241293265 for Wrist Stabilising Exercise Versus Hand Orthotic Intervention for Persons with Hypermobility – A Randomised Clinical Trial by Lindholm Susanne and Claesson Lisbeth in Clinical Rehabilitation
