# Supplementary material for: Wrist Stabilising Exercise Versus Hand Orthotic Intervention for Persons with Hypermobility – A Randomised Clinical Trial
Source: Clin Rehabil. 2024 Oct 29;39(1):47–57. doi: 10.1177/02692155241293265 (PMC11776353; doi:10.1177/02692155241293265)
Supplement: sj-docx-1-cre-10.1177_02692155241293265 - Supplemental material for Wrist Stabilising Exercise Versus Hand Orthotic Intervention for Persons with Hypermobility – A Randomised Clinical Trial [file sj-docx-1-cre-10.1177_02692155241293265.docx]

# Supplementary Material 1

Patient information

Static strength

training programme

for the wrist


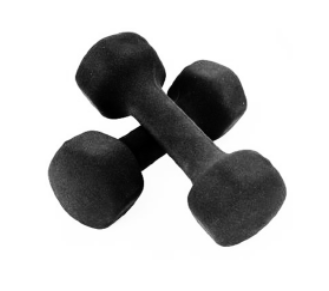


Hand and plastic surgery clinic

The rehabilitation unit

Tel. 010-103 18 87

Sit comfortably, relaxed in the arm and shoulder. Let the entire forearm rest against the table, keep the wrist outside the edge of the table. All exercises are performed with a straight wrist.

Exercise once a day. Do exercises 1-4 in 3 sets and then increase by one set per week up to 8 sets. Then increase the weight by ½ kilogram and start over from the beginning.

Starting weight:...................................

1. Hold the weight in your hand with the back of your hand facing upwards.

Hold the position for 10 seconds.


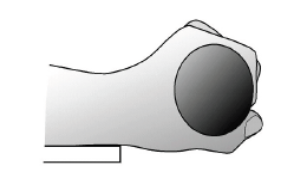


2. Hold the weight in your hand with the thumb side facing up.

Hold the position for 10 seconds.


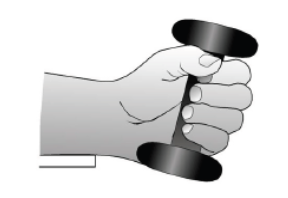


3. Hold the weight in your hand with the palm facing upwards.

Hold the position for 10 seconds.


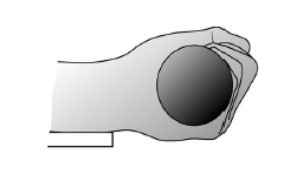


4. Support the forearm with the help of the other hand with the little finger side facing

upwards.

Hold the position for 10 seconds.


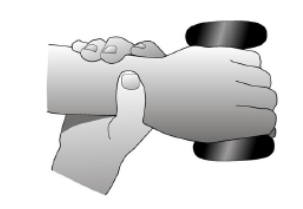


For heavier weights over 2 kilograms, exercise every other day.

Squeeze the ball as hard as you can without pain. Hug the ball 10 repetitions x 3 sets, then gradually increase by one repetition per week.


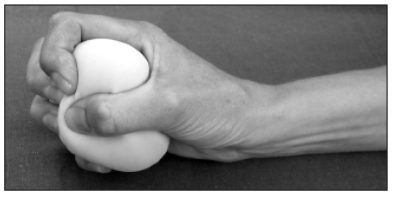


**Information**

- The ligaments are stretched or damaged and then it is the muscles that hold the structures/small bones in place in the wrist.
- Remember, when the wrist is in external position, the muscles cannot hold the small bones in the hand in place = PAIN!

**Avoid**

- Maximal extreme positions in connection with load, for example standing up and push-ups with a bent wrist.
- Monotonous movements.

**Council & regime**

- Always lifting things with straight wrists and with tense forearm muscles.
- Keep upper arm close to torso when lifting = short lever arm


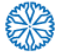
Ostergotland

County Council

Revised 2012-09-19.
