# Supplementary material for: Wrist Stabilising Exercise Versus Hand Orthotic Intervention for Persons with Hypermobility – A Randomised Clinical Trial
Source: Clin Rehabil. 2024 Oct 29;39(1):47–57. doi: 10.1177/02692155241293265 (PMC11776353; doi:10.1177/02692155241293265)
Supplement: sj-docx-2-cre-10.1177_02692155241293265 - Supplemental material for Wrist Stabilising Exercise Versus Hand Orthotic Intervention for Persons with Hypermobility – A Randomised Clinical Trial [file sj-docx-2-cre-10.1177_02692155241293265.docx]

# Supplementary Material 2

Patient information

Dynamic wrist

strength training programme


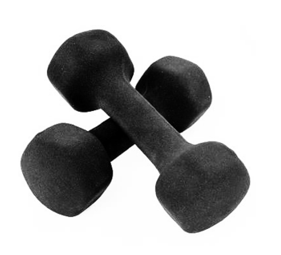


Hand and plastic surgery clinic

The rehabilitation unit

Tel. 010-103 18 87

Sit comfortably, relaxed in the arm and shoulder. Let the entire forearm rest against the table, keep the wrist outside the edge of the table. All exercises are performed with a straight wrist.

Increase the weight by ½ kilogram once per month.

ATTENTION! Perform the exercises in a pain-free range of motion.

Starting weight:................................... Number of repetitions/set: ..........................

1. Hold the weight in your hand with the back of your hand facing upwards.

Bend your wrist down and up.


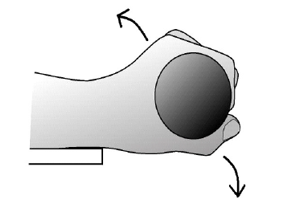


2. Hold the weight in your hand with the thumb side facing up.

Bend your wrist down and up.


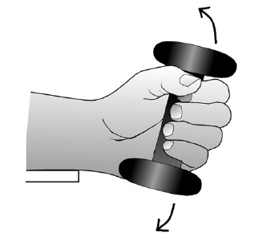


3. Hold the weight in your hand with the palm facing upwards.

Bend your wrist down and up.


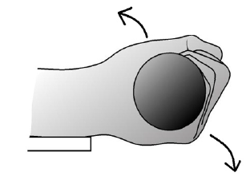


4. Support the forearm with the help of the other hand with the little finger side facing

upwards.

Bend your wrist down and up.


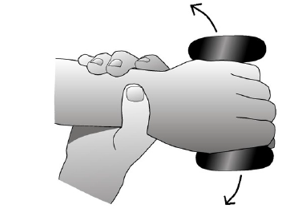


For heavier weights over 2 kilograms, exercise every other day.

Squeeze the ball as hard as you can without pain. Hug the ball 10 repetitions x 3 sets, then gradually increase by one repetition per week.


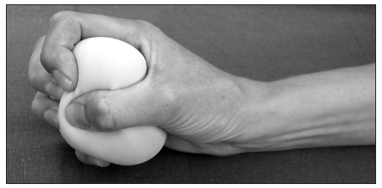


**Information**

- The ligaments are stretched or damaged and then it is the muscles that hold the structures/small bones in place in the wrist.
- Remember that when the wrist is in external position, the muscles cannot hold the small bones in the hand in place = PAIN!

**Avoid**

- Maximal external positions in in the wrist in connection with load, for example standing up and push-ups with a bent wrist.
- Monotonous movements.

**Council & regime**

- Always lifting things with straight wrists and with tense forearm muscles.
- Keep upper arm close to torso when lifting = short lever arm.


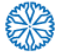
Ostergotland

County Council

Revised 2012-09-19.
